# A Thorough Review of Emerging Technologies in Micro- and Nanochannel Fabrication: Limitations, Applications, and Comparison

**DOI:** 10.3390/mi15101274

**Published:** 2024-10-21

**Authors:** Koosha Karimi, Ali Fardoost, Nikhil Mhatre, Jay Rajan, David Boisvert, Mehdi Javanmard

**Affiliations:** Department of Electrical and Computer Engineering, Rutgers University, Piscataway, NJ 08854, USA; koosha.karimi@rutgers.edu (K.K.); fardoost.ali@rutgers.edu (A.F.); nam227@scarletmail.rutgers.edu (N.M.); jkr85@scarletmail.rutgers.edu (J.R.); dsb228@scarletmail.rutgers.edu (D.B.)

**Keywords:** microchannels, nanochannels, photolithography, soft lithography, electron beam lithography, focused ion beam, wet/dry etch, 3D printing, laser micro-machining, injection molding, micro CNC

## Abstract

In recent years, the field of micro- and nanochannel fabrication has seen significant advancements driven by the need for precision in biomedical, environmental, and industrial applications. This review provides a comprehensive analysis of emerging fabrication technologies, including photolithography, soft lithography, 3D printing, electron-beam lithography (EBL), wet/dry etching, injection molding, focused ion beam (FIB) milling, laser micromachining, and micro-milling. Each of these methods offers unique advantages in terms of scalability, precision, and cost-effectiveness, enabling the creation of highly customized micro- and nanochannel structures. Challenges related to scalability, resolution, and the high cost of traditional techniques are addressed through innovations such as deep reactive ion etching (DRIE) and multipass micro-milling. This paper also explores the application potential of these technologies in areas such as lab-on-a-chip devices, biomedical diagnostics, and energy-efficient cooling systems. With continued research and technological refinement, these methods are poised to significantly impact the future of microfluidic and nanofluidic systems.

## 1. Introduction

The development and fabrication of micro- and nanochannels have significantly advanced in recent years, driven by the growing need for miniaturized systems across various fields such as biomedical research, environmental monitoring, material science, and industrial applications [1,2,3,4,5]. These microfluidic systems enable the precise manipulation of fluids at the microscale, offering profound benefits in terms of cost efficiency, material usage, and experimental control [6,7]. Microfluidics, as a multidisciplinary field, harnesses the properties of fluid flow in confined spaces to create microchannel networks that support a wide range of applications, including lab-on-a-chip (LOC) devices, biosensing applications, microreactors, and micro-scale heat exchangers [8,9,10].

Microchannels, typically fabricated from materials such as polydimethylsiloxane (PDMS), silicon, glass, and thermoplastics like PMMA, offer unique advantages due to their ability to handle small fluid volumes while providing enhanced control over physical processes [11,12,13,14]. The manipulation of these fluids at the microscale leverages phenomena like laminar flow, rapid diffusion, and thermal transport, which are distinct from fluid behavior at the macroscale [15,16]. These characteristics make microchannels indispensable for biosensing, drug delivery, disease diagnostics, and other biomedical applications. The versatility and scalability of microchannel fabrication have also made them critical in the design of devices used in chemistry, physics, and engineering [17,18,19].

Historically, traditional fabrication methods such as photolithography and soft lithography have been the cornerstone of micro- and nanochannel production [17,18]. Photolithography, widely employed in the semiconductor industry, relies on the transfer of a pattern onto a photosensitive material using light exposure through a mask. While this method has proven effective in producing high-resolution features, its limitations, such as cost and complexity, have spurred the exploration of alternative techniques [20]. Soft lithography, using elastomeric materials like PDMS, allows for more flexible and cost-effective microchannel production, particularly in rapid prototyping scenarios. However, despite its versatility, soft lithography is often constrained by lower resolution and challenges in creating highly complex or high aspect-ratio structures [21,22].

Emerging technologies such as 3D printing, electron beam lithography (EBL), focused ion beam (FIB) milling, and laser micromachining have been introduced as modern alternatives to overcome the limitations of traditional methods [23,24,25]. These techniques bring new levels of precision and scalability to micro- and nanochannel fabrication. For instance, 3D printing has revolutionized the field by enabling rapid, customizable, and cost-efficient fabrication of microfluidic devices with complex geometries [26,27]. This method allows the creation of intricate channel structures that would be difficult to achieve through conventional lithographic techniques. Similarly, electron beam lithography and focused ion beam milling offer ultra-high-resolution capabilities down to the nanoscale, making them ideal for applications that demand tight control over channel dimensions and fluid flow [28,29].

Laser micromachining has also gained traction due to its ability to process a wide range of materials, from polymers to metals, without the need for a cleanroom environment. This technique uses high-powered lasers to precisely etch micro and nanochannels, offering a fast and flexible fabrication process that can be tailored to various materials and device requirements [30,31]. Additionally, advances in injection molding techniques have paved the way for the high-volume production of microfluidic devices, particularly in industrial applications where scalability is a priority [32].

The choice of fabrication technique is often dictated by the specific requirements of the application, with factors such as resolution, cost, speed, and material compatibility playing critical roles [1,17,32]. As the field continues to evolve, there is a growing trend toward hybrid approaches that combine multiple fabrication techniques to achieve the desired channel geometries and material properties [32]. For example, researchers have integrated 3D printing with micromachined laser lamination to create complex transparent microfluidic devices. This approach eliminates the necessity for costly and time-consuming cleanroom environments while enhancing the design accuracy of the lamination process [33].

Moreover, the fabrication of micro- and nanochannels has extended beyond biomedical applications to areas such as energy conservation and environmental protection [34,35]. Microchannels are being incorporated into systems that enhance heat transfer in electronics [36], improve energy efficiency in solar-powered refrigeration systems [37], and even contribute to environmental clean-up by facilitating the removal of pollutants from industrial flue gases [38].

Despite the remarkable progress made in the field of micro- and nanochannel fabrication, several challenges remain. Scaling up these technologies for mass production, while maintaining the precision and cost-efficiency required for industrial applications, is a key focus of ongoing research. Additionally, regulatory and market adoption challenges, particularly in the biomedical sector, continue to pose obstacles to the widespread commercialization of microfluidic devices. However, with continuous advancements in fabrication technologies and the development of new materials, the potential applications of micro and nanochannels are poised to expand further, offering solutions to critical challenges in healthcare, energy, and the environment.

This paper aims to provide a comprehensive review of the latest advancements in the fabrication of micro- and nanochannels, focusing on both traditional and emerging technologies. The review will highlight the key advantages, limitations, and future prospects of each technique, offering insights into the evolving landscape of microfluidic device fabrication. By examining the most recent developments in photolithography, soft lithography, electron beam lithography, focused ion beam milling, wet and dry etching, 3D printing, laser micromachining, injection molding, and micro CNC, this review seeks to contribute to the growing body of knowledge in the field and pave the way for future innovations.

## 2. Fabrication Methods for Micro- and Nanochannels

### 2.1. Photolithography

Photolithography is a widely utilized and well-established method, particularly in the semiconductor industry, and is also employed for the creation of metal nanoparticles. Due to its widespread use, only a brief summary will be provided. The process works by transferring a pattern from a lithographic mask onto a light sensitive resist layer, which is pre-applied to a substrate [39].

**Figure 1:**
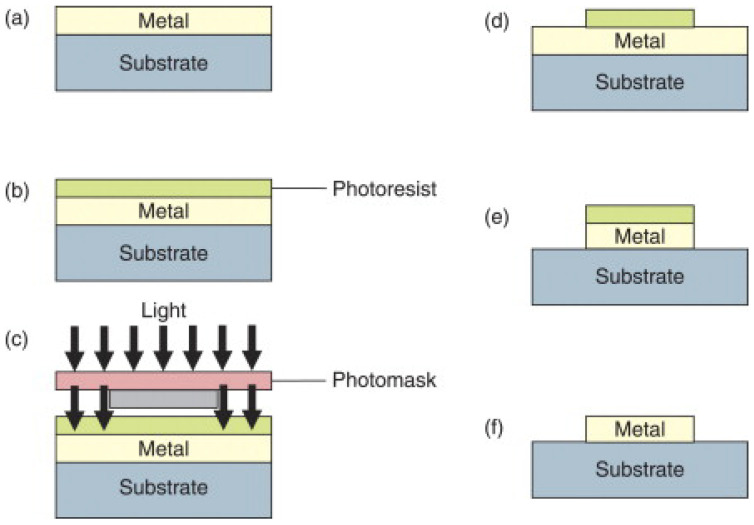
Photolithography technique steps using positive resist. (**a**) Metal deposited on the substrate. (**b**) Photoresist is spin-coated on the sample. (**c**) Light exposure through a mask for patterning the structure. (**d**) Developing the photoresist. (**e**) Etching the unwanted metal surface. (**f**) Resist removal. Reprinted with permission from Ref. [39]. 2011, Elsevier.

There are two primary options: using a positive resist or a using negative one. The typical procedure for a positive resist is shown schematically in Figure 1. Initially, a thin metal film is deposited onto the substrate (Figure 1a). Following this, a photoresist is spin-coated onto the sample, and a soft baking process is carried out (up to 30 min at temperatures ranging from 60 to 100 °C) (Figure 1b). The sample is then exposed to light through a mask (Figure 1c), transferring the mask’s design onto the resist, where photochemical changes occur. Afterward, the resist is developed and undergoes hard baking for 20–30 min at temperatures between 120 and 180 °C (Figure 1d). The unprotected metal areas are etched away (Figure 1e), and finally, the remaining resist is removed, leaving the metal nanoparticles on the substrate (Figure 1f) [39].

**Figure 2:**
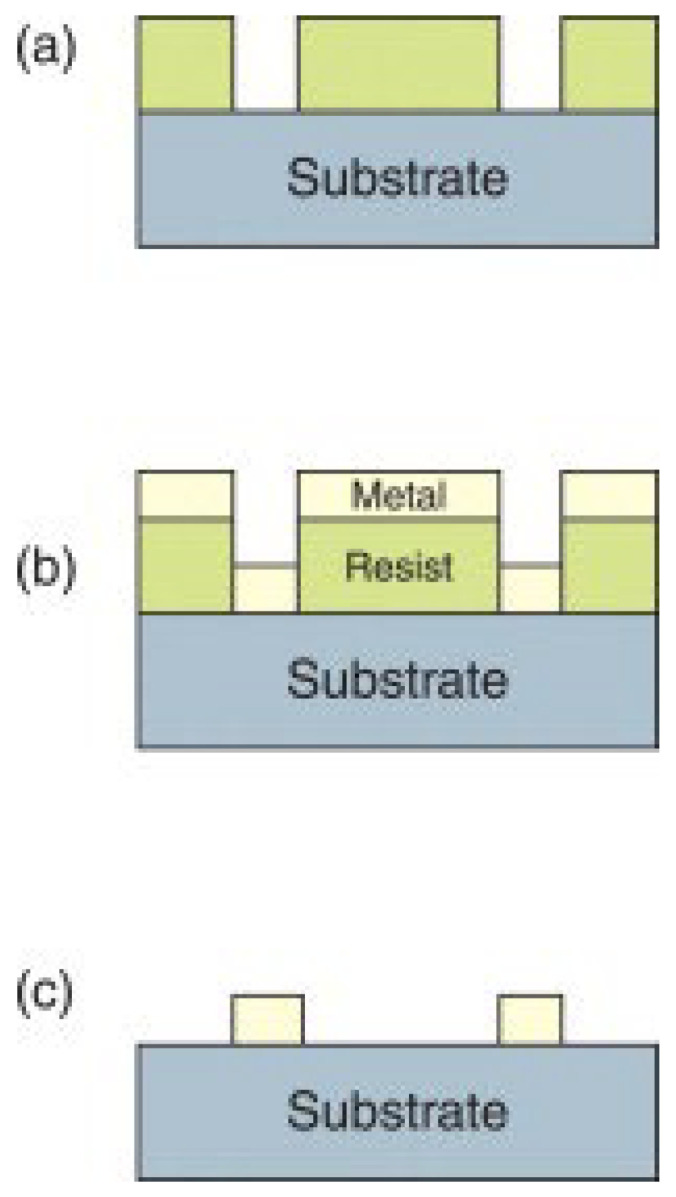
Photolithography technique steps using negative resist. (**a**) The photoresist is first deposited onto the substrate and then undergoes a soft bake. Next, it is exposed to light through a mask, developed, and followed by a hard bake, leading to the formation of void areas. (**b**) Metal deposition leads to filling the void areas. (**c**) Residual resists and metal deposited on the top of them are chemically removed. Reprinted with permission from Ref. [39]. 2011, Elsevier.

For a negative resist, the procedure is largely similar to the one shown in Figure 1. When fabricating nanoparticles with negative resist, the resist is directly deposited onto the substrate and soft baked. Afterward, the resist is exposed through a mask, developed, and hard baked. This creates empty regions, as shown in Figure 2a, which are subsequently filled by depositing a metal layer onto the sample (Figure 2b). Finally, the remaining resist, along with the metal deposited on top, is removed chemically, leaving the metal nanostructures (Figure 2c) [39].

The main advantage of photolithography is its capability to support mass production through parallel processing. However, it is constrained by the Abbe–Rayleigh criterion [40,41,42]:

$$d_{min}=\frac{0.61\lambda }{n\sin\alpha }=\frac{0.61\lambda }{NA}$$

where *d_min_* represents the smallest possible feature size, *λ* is the wavelength of the light used, *n* is the refractive index of the surrounding environment, *α* is the aperture angle, and *NA* is the numerical aperture of the system.

For finer structures, higher numerical apertures and shorter wavelengths are necessary. A high-pressure mercury lamp is sufficient for structures larger than 250 nm, but for feature sizes between 150 and 250 nm, shorter wavelengths—such as those produced by excimer lasers (193 nm for ArF; 248 nm for KrF)—are required. For features below 100 nm, light wavelengths shorter than 150 nm must be employed. Extreme ultraviolet (EUV) lithography, which operates at wavelengths as short as 13.4 nm, is one approach [38]. Wu and Kumar have reviewed EUV lithography in detail [43].

Another drawback of photolithography is that the side walls of the produced structures are typically perpendicular to the substrate surface. While the lateral shape of the structures can be freely designed, the profile of the side walls cannot. Additional steps are needed to overcome this limitation [39]. For example, Kontio et al. developed conical metallic nanostructures by using a two-step lithographic process, leveraging the shadowing effect during metal deposition [44].

Some notable novel research in photolithography includes the work of Yin et al. and Lee et al. Yin et al. developed a cost-efficient method for fabricating two-dimensional silicon nano molds for linear nanochannels, achieving sub-100 nm channels on 4-inch wafers using UV photolithography, oblique copper nano-mask deposition, sputter etching, and photoresist/copper removal. Their technique enables precise channel width control by adjusting the deposition angle and photoresist height [45]. Moreover, Lee et al. fabricated hybrid Polydimethylsiloxane (PDMS) channels with nano/microchannels and 3D microfunnels using dual PDMS layer casting, PDMS/glass bonding, two-step UV lithography, and one-step pyrolysis. Their innovation achieved 90% volume shrinkage, enabling carbon nano-mold fabrication and the precise alignment of 3D structures without complex nanofabrication [46].

### 2.2. Soft Lithography

**Figure 3:**
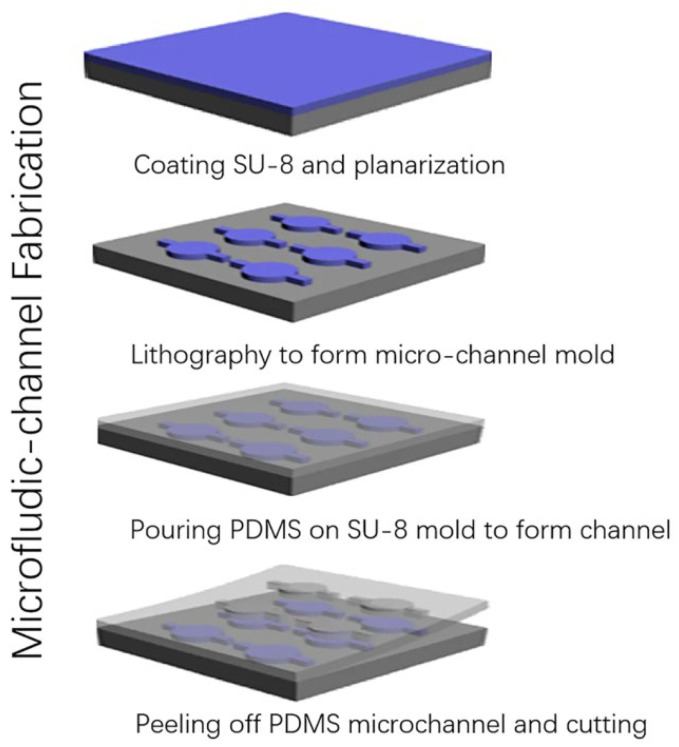
The process of fabricating the microfluidic channels employed in a THz biosensor using the replication method of the soft lithography technique [48].

Soft lithography is a technique used to shape soft materials and create micro- and nanochannels. It involves creating precise patterns on a substrate using polydimethylsiloxane (PDMS) as a stamp mold, which can be used at least 50 times without being altered [47]. Figure 3 depicts the process of fabricating the microfluidic channel made of PDMS utilized in a THz biosensor using replication technology and SU-8 molds created through photolithography. Note that preventing surface contamination is crucial during fabrication processes. Prior to fabrication, PDMS and SU-8 should be prepared in varying ratios. The process begins with spinning a 60 μm-thick SU-8 layer onto a polished silicon wafer, followed by soft baking. The desired features, including the microchannel, inlet, and outlet, are patterned onto the SU-8 layer via standard photolithography. Post-exposure baking and development are then conducted, followed by rinsing with isopropyl alcohol to remove any residual SU-8. The mold is baked to improve adhesion between the SU-8 and the silicon wafer. Prior to PDMS casting, the surface is treated with a mold release agent to allow for repeated use. Liquid PDMS is then cast onto the mold, cured, and carefully peeled from the substrate to obtain the microchannel structure [48]. Some established methods for soft lithography include MIMIC, replica molding, and embossing. However, these methods are time and cost-consuming, resulting in low throughput [49]. Once the PDMS mold is fabricated, it provides an efficient way to alter cells, mediate cell capture, and grow stem cells [50]. Although PDMS is widely used, it can be further improved with surface modifications such as nanowires and nanoparticles to increase electrical conductivity [51]. While the traditional methods rely on a permanent mold, the technology has been evolving, and reconfigurable molds are now being used to generate the channels.

One method called self-forming masters involves curing a liquid layer into a film and, then, pressing a stamp into the film to create patterned surfaces [49]. This method is suitable for flexible structures and is used in applications such as functional transistors, capacitors, and LED devices [52]. Natural materials, like rose petals and butterfly wings, can also be used in lithography to create intricate details. By using the negative of the surface, a master mold is created and implemented in the soft lithography process at a low cost. This provides a unique output while still using the established methods of pouring liquid elastomer over the nanostructure and then removing the piece to make the mold. In addition, 3D printed soft lithography uses a 3D model as a template to create a negative mold. Silicone elastomer is filled into the mold and cured, allowing the production of multiple replicas of the 3D printed model. The silicone replica is then bonded to a glass substrate to create a final microfluidic device [53]. This method provides flexibility when developing a mold for PDMS but requires expensive instrumentation and a clean room setting for proper implementation [54,55].

The application of soft lithography to create microchannels has yielded innovative possibilities owing to its straightforward methodology. Notably, the rapid assembly of a multi-layer organ on a chip device has been achieved through the utilization of PDMS and machining. This process encompasses three primary stages: design, machining, and assembly, where xurography is integrated. Remarkably, xurography, involving the use of a cutting plotter to trim thin film plates, distinguishes this approach [56]. Furthermore, the use of soft lithography has facilitated the development of a microbial fuel cell incorporating PCB electrodes and a soft lithographic microchannel. The resulting chip has exhibited commendable performance, delivering stable power for 70 h and displaying ease of reproducibility. The soft lithography process has notably enhanced the fuel cell’s performance by enabling the optimization of microchannel layouts to facilitate the flow of fuel and oxidants within the cell [57]. While soft lithography stands as a meticulously researched technique for crafting microchannels, contemporary methods and applications continue to draw from the foundational principles of the original approach.

Soft lithography using PDMS, while flexible and cost-effective, faces limitations when fabricating high aspect-ratio structures or intricate designs like those required in advanced microfluidic devices. These limitations can be mitigated by combining soft lithography with other techniques or by employing surface modification strategies. One approach to enhance resolution and overcome the aspect-ratio challenge is to combine soft lithography with reactive ion etching (RIE). RIE improves the etching precision by offering anisotropic etching profiles, allowing the creation of smoother, more detailed structures. Oxygen plasma treatment of PDMS surfaces prior to RIE can significantly improve the adhesion of photoresists, enabling high-resolution features to be transferred to the PDMS layer. However, despite its potential, the process is often hampered by the difficulty in maintaining high etch rates and controlling surface roughness during etching, as PDMS is not inherently compatible with conventional photolithography techniques designed for semiconductor materials [58,59]. Another promising approach would be a surface modification method involving sol-gel coating, which can create a thin, glass-like oxide layer on the PDMS surface. This technique not only improves the mechanical and chemical resistance of the microchannels but also helps prevent issues like diffusion and swelling, which are common in PDMS-based devices. This surface modification can be further functionalized for specific applications by using different silane reagents. In contrast to other methods, sol–gel coatings offer longer-lasting hydrophilicity, which is crucial for maintaining functional microfluidic channels [59,60]. These strategies illustrate that combining other lithographic techniques with soft lithography or performing surface modifications opens up possibilities for creating highly detailed and robust microstructures in PDMS, addressing some of the key limitations of soft lithography in microfluidic device fabrication.

### 2.3. Electron Beam Lithography (EBL)

**Figure 4:**
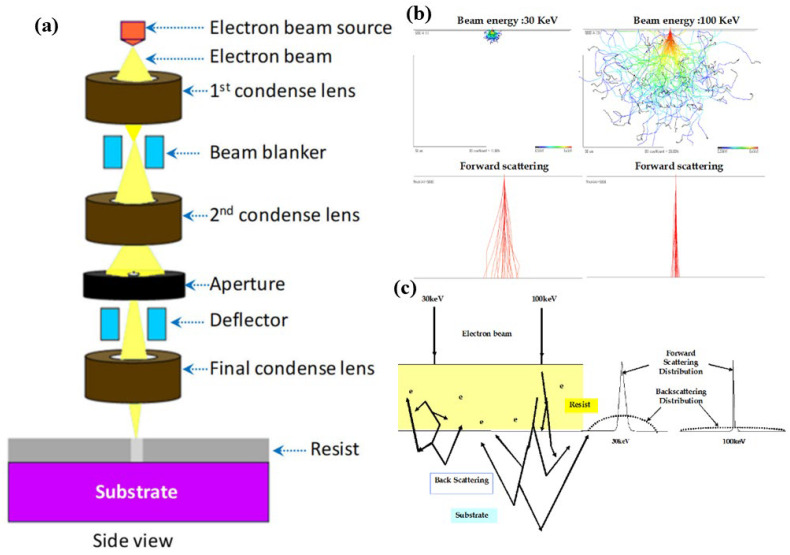
(**a**) E-beam lithography mechanism. EBL consists of a chamber, an electron gun, and a column. The column and chamber are maintained under a high vacuum. The column houses all the electron-optical components responsible for generating, accelerating, controlling (turning on and off), focusing, and deflecting the electron beam according to the writing pattern. In the main chamber, samples are typically loaded via a load lock and placed on an interferometric stage to ensure precise workpiece positioning [65]. (**b**) Simulation of electron scattering trajectories on a resist-coated silicon substrate at 30 kV and 100 kV incident energies. (**c**) Electron-scattering behavior for electron beam energies of 30 keV and 100 keV on a resist-coated silicon substrate. When an electron beam strikes the resist, many electrons undergo small-angle forward scattering, which broadens the primary beam. As the electrons penetrate deeper into the resist and substrate, some experience large-angle scattering, resulting in backscattering. These backscattered electrons can re-enter the resist at locations far from the primary beam’s entry point. Also, forward scattering is caused by electron–electron interactions that slightly deflect the primary electrons, leading to beam broadening in both the resist and the substrate [66].

Electron-beam lithography (EBL) is a pivotal technology in the field of nanofabrication, renowned for its ability to achieve high resolution, reliability, and precision [61]. Unlike conventional photolithography, which utilizes UV light to transfer patterns onto a substrate, EBL employs a focused beam of electrons. This allows for the fabrication of intricate nanoscale structures with feature sizes as small as 2–10 nanometers, a critical factor in the fabrication of both micro- and nanochannels. As these channels often require tight dimensional control, EBL provides a highly accurate method for creating the necessary patterns [62,63]. The process of EBL begins with the deposition of an electron-sensitive resist, typically, polymethyl methacrylate (PMMA), on the substrate. The electron beam is then used to scan the resist in a desired pattern, modifying its solubility in a developer solution. Depending on the type of resist—positive or negative—the exposed regions will either become more or less soluble. After exposure, the resist is developed, selectively removing the patterned areas. These patterns can then be transferred to the underlying substrate through subsequent etching or deposition steps [64]. Figure 4a shows the mechanism of the electron beam lithography.

EBL offers several advantages, particularly in its ability to achieve ultra-high resolution. With features down to the sub-10 nm range, it is well-suited for creating nanoscale channels that require extreme precision in their geometry. The technique is also highly flexible; unlike optical lithography, which relies on pre-made photomasks, EBL is maskless, and pattern design can be easily modified through computer control [67,68]. This flexibility makes EBL especially valuable in research and development settings, where frequent design changes are common. Additionally, EBL is versatile and can be used with various substrates and materials, further broadening its application potential for micro- and nanofluidic devices [69]. However, EBL is not without its limitations. A significant drawback is its low throughput. Since EBL is a serial process—where the electron beam writes patterns point-by-point—it is considerably slower than parallel techniques like photolithography. This makes EBL less suitable for mass production, especially over large surface areas. Another challenge is the proximity effect, where electron scattering in the resist and substrate causes unintended exposure in adjacent areas, potentially reducing pattern fidelity [70]. This challenge may diminish the resolution and precision in fabricating intricate nanochannel networks. Future research could focus on investigating proximity correction methods or developing more advanced resist materials to address this issue. Figure 4b,c illustrate the electron scattering in electron beam lithography. Furthermore, EBL systems are expensive, both in terms of the equipment required and operational costs, adding to their limitations, especially in industrial-scale applications [71]. However, recent advances in EBL technology aim to address some of the method’s limitations. For instance, the development of multi-beam EBL systems seeks to enhance throughput by enabling parallel patterning. Additionally, innovations in resist materials, including the use of inorganic resists, have led to improved resolution and etch resistance. These developments push the boundaries of EBL, making it even more relevant for the fabrication of the next generation of micro- and nanochannels [70,72,73].

In the context of micro- and nanochannel fabrication, EBL’s high resolution and patterning capabilities make it indispensable. The technique allows for creating complex, multi-dimensional channels essential for applications such as microfluidics, lab-on-a-chip systems, and biomedical diagnostics [74,75,76]. Nanochannels created using EBL offer fine control over fluid flow and other physical processes, making them suitable for precision applications in fields ranging from drug delivery to chemical analysis [77,78].

### 2.4. Focused Ion Beam (FIB) Milling

**Figure 5:**
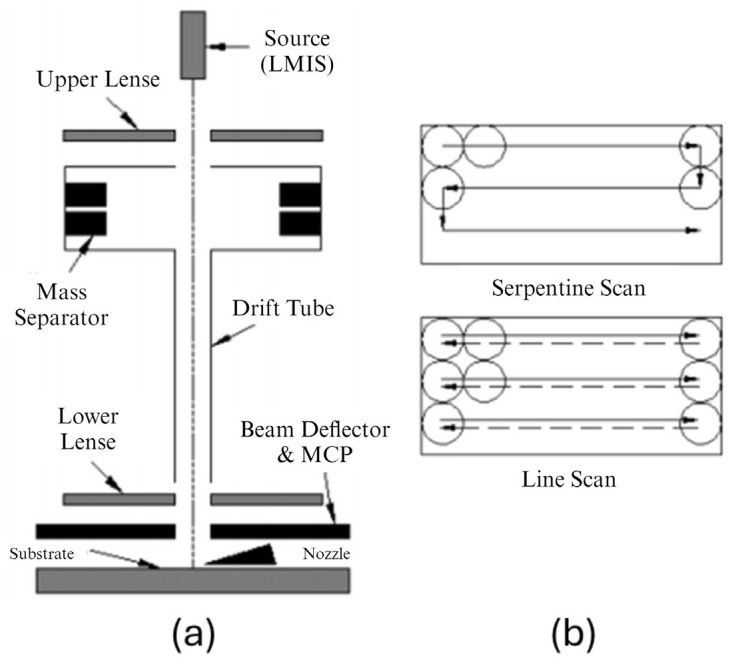
(**a**) A two-lens FIB system and (**b**) serpentine and line scanning of a rectangular pattern [82].

Focused ion beam (FIB) milling is a highly specialized technique utilized in the fabrication of micro- and nanoscale structures. It operates by directing a concentrated beam of ions, typically gallium, onto a target material, where the ions displace surface atoms and etch or mill away layers in a highly controlled manner. The key components of a typical FIB system include an ion source, an ion optics column, a beam deflector, and a substrate stage [79]. Figure 5a presents a schematic of a FIB system with a two-lens (or twin-lens) column. The liquid metal ion source (LMIS) is commonly used to generate consistent and stable ion beams for various ion species [80,81], with Ga^+^ ions being the most frequently used. The application of the critical Taylor voltage to the liquid metal cone extracts positively charged ions, forming the ion beam. The full width at half maximum (FWHM) is typically used to describe the diameter of the FIB, where the ion intensity is distributed non-uniformly, often resembling a Gaussian profile. FWHM is defined as the distance between points on the intensity profile where the intensity reaches half of its maximum value [82,83]. In FIB milling, the Ga^+^ ion beam can be digitally raster-scanned in a serpentine pattern across the rectangular area to be milled, or it can be line-scanned along a line, which is then systematically stepped to cover the rectangular region, as illustrated in Figure 5b [82].

This method offers exceptional precision, with features being milled at resolutions down to the nanometer scale, making it indispensable in areas like semiconductor manufacturing, advanced microscopy sample preparation, and nanotechnology. FIB milling is particularly valued for its ability to produce complex geometries and intricate 3Dstructures that would be difficult or impossible to fabricate using conventional lithographic or subtractive methods. By utilizing a focused beam, the material can be removed layer by layer, allowing for the creation of finely detailed patterns and devices. According to Tseng (2004), the FIB process is not only ideal for precise milling but also for patterning, implantation, and deposition applications, which enhances its versatility in various research and industrial applications [83].

In the field of semiconductor device fabrication, FIB milling is critical for circuit editing, defect analysis, and the creation of prototype components. Since the ion beam can be finely controlled, FIB milling is often used to modify integrated circuits or to repair photomasks in semiconductor fabrication. Additionally, its high resolution and direct write capability make it suitable for the creation of nanostructures and nanopatterns, which are essential for modern electronics and optical devices. This technology is also extensively used in the preparation of samples for transmission electron microscopy (TEM) and atom probe tomography (APT), where thin, defect-free specimens are essential for achieving high-resolution imaging. As discussed by Larson et al. (1999), FIB milling enables precise thinning of samples down to the atomic level, making it an indispensable tool for materials scientists who require accurate cross-sections or thin specimens [84].

Beyond its applications in the semiconductor industry, FIB milling is widely employed in materials science for microfabrication of tools and devices. Micro- and nanotools created using FIB technology have applications in precision machining, particularly in producing cutting tools with nanometer-scale sharpness. These tools are used in ultraprecision lathes for processing metal alloys, ceramics, and other materials with a high degree of accuracy. In their review, Bhavsar et al. (2009) illustrate how FIB milling enables the creation of microtools with tailored shapes, such as needles and probes, which are used in both mechanical machining and biological applications like micro-injection and nanomanipulation [82].

**Figure 6:**
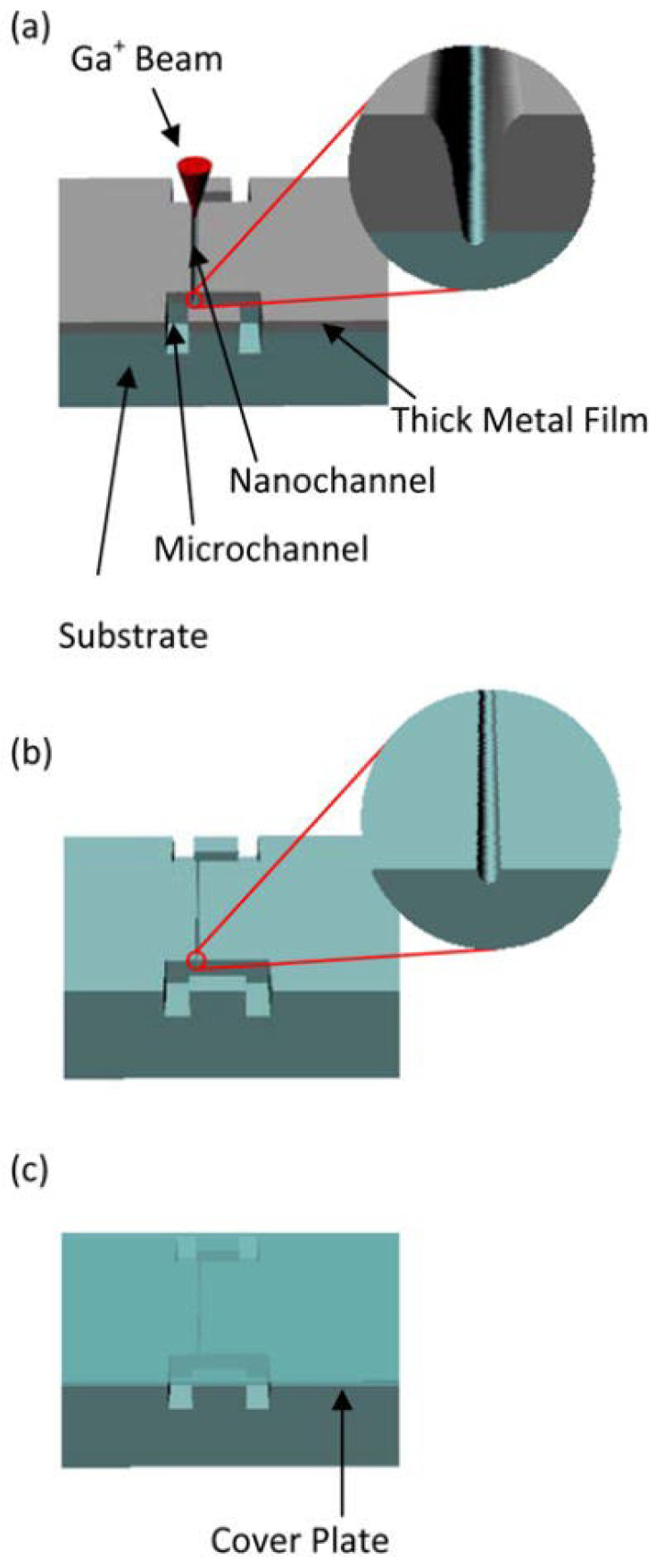
FIB milling process and succeeding fabrication steps schematic. (**a**) A nanochannel is milled through the thick metal film. (**b**) The metal film is removed using an etching solution. (**c**) Micro- and nanochannels are sealed with a cover plate [85].

Another important application of FIB milling is in the fabrication of nanopores, nanochannels, and other nanostructures. These structures are critical for biological sensing, molecular analysis, and lab-on-chip devices. Nanopores fabricated using FIB can be used for DNA sequencing and the detection of biomolecules, as their small dimensions allow for the passage of individual molecules through the pore, which can be detected and analyzed. As portrayed in Figure 6, Menard and Ramsey (2011) describe the use of FIB milling to create sub-5 nm nanochannels in insulating substrates, which are crucial for applications in nanofluidics and molecular transport studies [85]. These nanochannels, fabricated with high precision thanks to the FIB milling technique, are essential for ensuring controlled flow and detection of nanoscale particles, opening new possibilities in fields such as biophysics and nanomedicine.

### 2.5. Etching Techniques (Wet and Dry Etching)

Etching is a fundamental process in fabricating micro- and nanochannels, allowing for the precise removal of material to define patterns or structures. Two main etching techniques are commonly employed: wet etching and dry etching. Each method has distinct characteristics that make it suitable for specific fabrication needs. Understanding their mechanisms, advantages, and limitations is crucial when deciding the appropriate technique for micro- and nanoscale channel fabrication [86].

Wet etching involves the use of chemical solutions to dissolve material from a substrate. This process is often isotropic, meaning that material is etched uniformly in all directions, though anisotropic etching can be achieved under specific conditions. One of the key advantages of wet etching is its simplicity and cost-effectiveness, as it requires relatively basic equipment and offers high etching rates. Wet etchants are highly selective and capable of removing certain materials while leaving others intact, making them useful for multilayered structures. Common wet etchants include potassium hydroxide (KOH) for silicon and hydrofluoric acid (HF) for silicon dioxide. However, a major limitation of wet etching is the potential for undercutting, as the isotropic nature of the process can cause unwanted material removal beneath the patterned areas. This limits its ability to create high-aspect-ratio features [87]. In the context of micro- and nanochannel fabrication, wet etching is frequently employed for etching sacrificial layers or creating large, shallow features. For example, in silicon microchannel fabrication, KOH is often used to create V-shaped grooves. This process is especially useful for applications in fluid transport where high precision is not a strict requirement. However, as device designs become more complex and require finer control over feature geometry, wet etching may not offer the necessary precision [88,89,90]. Wang et al. present a novel method for fabricating high-quality nanochannels using a fast, low-cost, and maskless approach based on friction-induced selective etching in HF/HNO_3_ mixtures. This technique is a significant improvement over traditional methods like photolithography, as it eliminates the need for masks and reduces fabrication complexity and cost. The study highlights how the anisotropic etching ability of HF/HNO_3_ mixtures is effectively harnessed to create precise nanochannels on silicon substrates, with parameters such as volume ratio, scratching load, and etching time systematically optimized for better control. The novelty lies in the use of friction-induced selective etching, which provides a straightforward, efficient, and scalable solution for producing nanochannels, making it highly suitable for applications in nanofluidics, analytical chemistry, and biochemistry [91].

**Figure 7:**
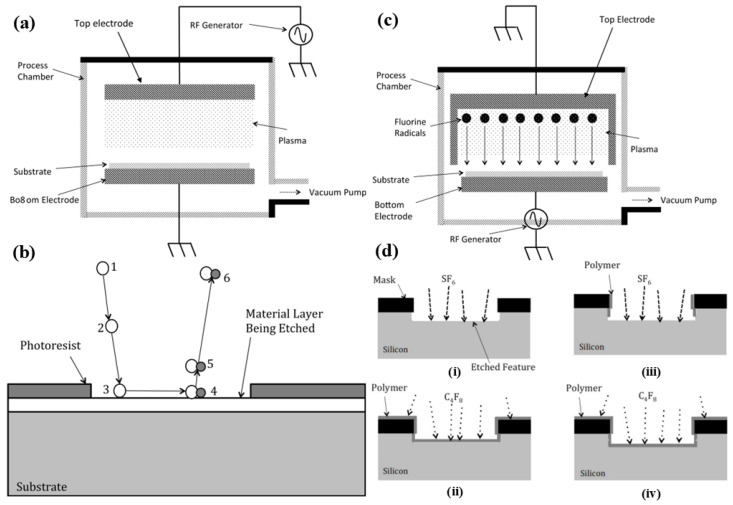
The schematic of three major methods for dry etching. (**a**) Diagram of a plasma etching system: The substrate is placed on the bottom electrode, which is electrically grounded, while the top electrode is connected to a radio frequency (RF) generator. (**b**) Diagram depicting the six stages of plasma etching: Step 1: Process gases are ionized into chemically reactive species within the plasma. Step 2: These reactive species diffuse toward the substrate surface. Step 3: Reactive species are absorbed onto the material layer. Step 4: A reaction occurs between the reactive species and the material layer. Step 5: The by-products of this reaction are desorbed. Step 6: The by-products diffuse away. (**c**) Diagram of a reactive ion etching (RIE) system. (**d**) Diagram illustrating the process mechanism for deep reactive ion etching (DRIE) of silicon. (i) SF6 is utilized to generate fluorine-based reactive species for silicon etching. (ii) The etch tool deactivates the SF6 gas and activates the C4F8 gas. (iii) C4F8 is then deactivated, and SF6 is reactivated. (iv) SF6 is turned off once more, and the polymerization gas C4F8 is reintroduced [92].

Dry etching, in contrast, is a process that removes material using ionized gases or plasma in a vacuum environment. Dry etching can be highly anisotropic, allowing for directional etching, which is ideal for creating deep, narrow, and well-defined micro- and nanochannels. There are several types of dry etching, including plasma etching, reactive ion etching (RIE), and deep reactive ion etching (DRIE). Figure 7 shows different dry etching techniques. Plasma etching relies on chemically reactive ions, while RIE combines chemical reactions with physical ion bombardment for greater control over etching depth and direction. DRIE is particularly important in the fabrication of high-aspect-ratio channels and is widely used in the production of microfluidic devices and MEMS (micro-electro-mechanical Systems) [92,93]. Dry etching offers superior precision and the ability to create complex geometries with vertical sidewalls, making it a valuable technique for advanced micro and nanochannel fabrication. However, it requires more sophisticated equipment and a controlled vacuum environment, making it more expensive and slower than wet etching. Despite the higher cost, dry etching’s versatility and precision make it indispensable in applications where accuracy and feature control are critical. For instance, DRIE processes using gases like sulfur hexafluoride (SF6) are commonly used to etch deep silicon trenches for fluidic applications [94,95].

### 2.6. 3-D Printing

Three-dimensional printing is an innovative manufacturing technique that allows the creation of biomedical devices, which were traditionally difficult to design using conventional methods such as machining or molding. Recently, there has been a growing interest in 3D-printed microfluidics due to its advantages, including fast production, cost-effectiveness, and precise fabrication of a wide range of complex products. This is made possible by new technologies such as stereolithography, digital light processing, fused deposition modeling, and inkjet printing, which can be used for the fabrication of micro- and nanochannels [96,97,98,99]. Three-dimensional printing is cost-effective due to its ability to minimize material waste through additive manufacturing [100], reduce prototyping costs by enabling rapid iteration without expensive molds or tooling [101], and handle complex designs without significant cost increases [102]. Additionally, it is scalable for small to medium production runs, making it ideal for custom or on-demand microchannel manufacturing without the need for specialized equipment [103].

**Figure 8:**
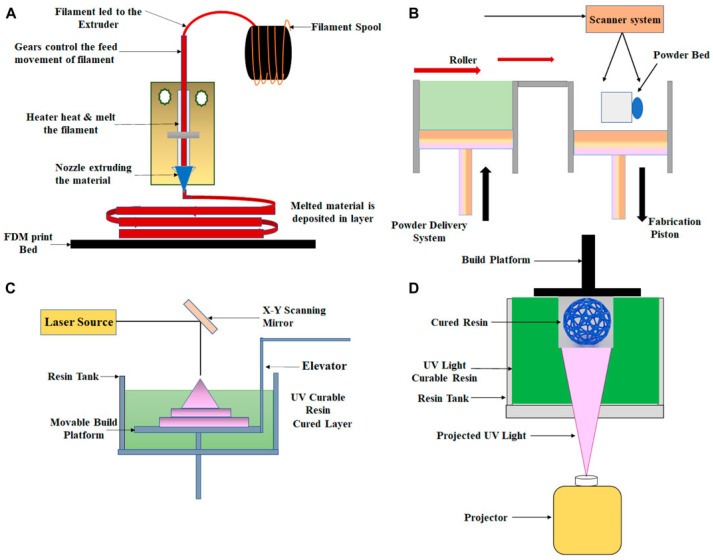
3D-printing techniques suitable for biomedical application. (**A**) Fused deposition modeling, (**B**) selective laser sintering, (**C**) stereolithography, and (**D**) digital light processing [99].

Fused deposition modeling (FDM) is a method that entails the extrusion of melted thermoplastic material layer by layer to achieve a desired shape. This process involves the propulsion of a polymer through a heated nozzle, followed by solidification through cooling, as illustrated in Figure 8A. FDM is esteemed for its user-friendly nature and cost-effectiveness. Moreover, this technique facilitates the production of optically transparent devices. A recent investigation has disclosed the potential of poly(methyl methacrylate) (PMMA) and acrylonitrile butadiene styrene (ABS) thermoplastic materials for the fabrication of optically detectable 3D-printed microfluidic devices [104]. Nonetheless, limitations are inherent in this technology. Due to the material’s extrusion through a nozzle, the production of small, intricately detailed items poses a considerable challenge. The resolution is contingent upon the size of the extruded filament, and the molten polymer necessitates time to solidify, rendering unsupported components within an enclosed channel susceptible to collapse [105].

Selective laser sintering (SLS) represents a 3D-printing technique that falls under the powder bed fusion category. In this process, a laser beam is employed to construct layered objects by melting and fusing the powder material. The consolidation of powder in SLS encompasses various particle-binding mechanisms, including chemical reactions, solid-state sintering, and partial or absolute melting. Controlled laser beam scanning is utilized to sinter the powder through heating. The resolution of the 3D object is contingent upon the particle size of the powder, scanning speed and spacing, laser power, and powder quality. The iterative repetition of the process results in the fabrication of the final 3D object. A schematic of the SLS is shown in Figure 8B. Common materials used in the SLS process include polymers such as polystyrenes (PS), thermoplastic elastomers (TPE), polyamide (PA), polyaryletherketones (PAEK), and polycaprolactone (PCL) [106,107].

Stereolithography, also referred to as SLA, is an additive manufacturing technique used for the creation of 3D objects. This process leverages a focused laser to solidify liquid photo-polymer resins, allowing for the precise formation of intricate structures. By employing photosensitive thermoset polymers in their liquid state, solidification occurs when a UV laser beam is directed to a specific point, as demonstrated in Figure 8C. This method enables the incremental layering of materials, facilitating the construction of complex shapes with exceptional precision. High surface resolution and precision are among the primary benefits of the SLA technique. Additionally, the production of intricate components at a minimal cost is achievable due to the limited use of liquid medium [99]. The widespread application of SLA extends to the manufacturing of microfluidic chips, featuring channels ranging from a few hundred micrometers to millimeters [108]. These versatile techniques are instrumental in the development of various devices, encompassing sensors, actuators, microfluidic devices, and biomedical implants. Furthermore, the utilization of SLA contributes to the creation of lab-on-a-chip devices for point-of-care diagnostics, drug delivery systems, and organ-on-a-chip models [99,109].

Digital light processing (DLP) is a similar technique to SLA that uses a projector to solidify the resin instead of a laser. Instead of using a layer with numerous laser scan routes, this method creates each 2D layer after the liquid polymer is safely exposed to the projector’s light, as shown in Figure 8D. DLP is utilized to create active component sensors that could change shape as needed. Prototypes with great resolution, reproducibility, and precision can be manufactured with this process [99]. One study shows that DLP can be used to make organs-on-a-chip (OOC). Typically, these are made using soft lithography and PDMS. However, in this study, researchers found that by using commercial PlasCLEAR resin and the PEG-DA-based mixtures, they were able to find a suitable product for the fabrication of transparent OOCs [110]. New technology like this allows researchers to properly observe what is happening within their OOCs and make proper conclusions.

Three-dimensional printing stands as a groundbreaking technology with the capacity to produce high-resolution microfluidics for the next generation. The inherent advantages of 3D printing lie in its customizable nature, the capability to fabricate intricate designs, and the efficient utilization of resources to minimize waste. This technology holds significant potential for the fabrication of microfluidic devices with applications in medical sensing and various industrial sectors. Furthermore, 3D printing empowers engineers to conceive more intricate and integrated microfluidic systems, thereby propelling the advancement of the next generation of microfluidic technologies [111,112,113]. The exploration of technologies such as stereolithography, digital light processing, fused deposition modeling, and selective laser sintering merely marks the initiation of high-speed and high-resolution 3D printing.

Three-dimensional printing fabrication methods can also be integrated with other methods for a more efficient fabrication process. Wu et al. recently explored a new method for fabricating microfluidic chips by integrating 3D printing with polymer dissolution technology. This approach uses poly(vinyl alcohol) (PVA) and high-impact polystyrene (HIPS) as channel molds that can be dissolved after encapsulation in PDMS, creating internal microchannels without requiring a separate bonding process. The novelty of this study lies in its method of forming microchannels that bypass complex alignment and bonding steps, thereby enhancing the stability and accuracy of microfluidic chip fabrication, especially when using HIPS as the channel material. This approach offers a simpler and more efficient solution for manufacturing microfluidic chips with improved channel quality [114].

### 2.7. Laser Micromachining

**Figure 9:**
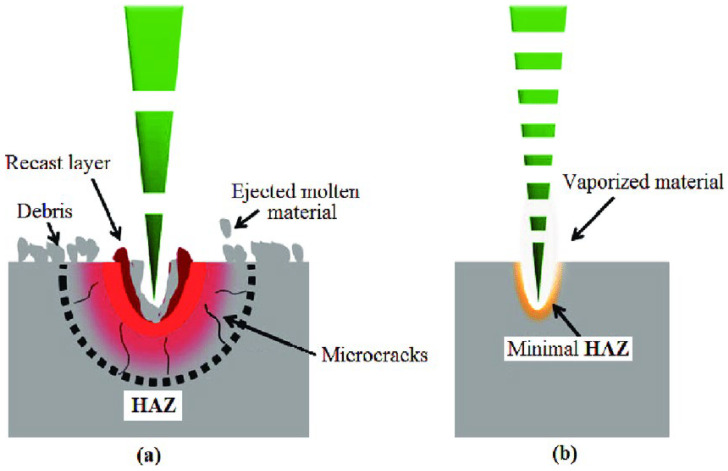
A comparison of how laser micromachining affects the targeted material with UV lasers versus IR lasers [118]. Clearly, it is observable that (**a**) A longer pulse duration (UV laser) increases the heat affected zone (HAZ) and leaves high thermal stresses resulting in crack and void formation, as well as surface debris. However, (**b**) short pulse duration (IR laser) leads to lesser thermal conduction thus resulting in precise machining operation and good surface finish [119]. This distinction is particularly important when dealing with sensitive materials like PDMS, PMMA, or glass, where maintaining surface quality is crucial. Employing shorter pulse durations or shifting to IR lasers can minimize thermal stress and enhance surface finish, making these methods more appropriate for high-precision microfluidic applications.

Laser micromachining offers cost-effectiveness, accelerated processing, enhanced flexibility, improved efficiency, and reduced residual waste, and does not necessitate a cleanroom or specialized personne; also, it is applicable to a wide range of materials used for microchannels, such as PMMA, PDMS, glass, and metal substrates [2,115,116]. The selection of laser type is contingent upon the properties of the chosen substrates. UV (ultraviolet) lasers can disintegrate a target substrate in brief pulses due to their high-energy illumination. However, these UV lasers are notably costly and less reproducible, posing challenges for consistent production at a commercial scale [115]. Conversely, IR (infrared) lasers offer a more economical option and have demonstrated significant success in processing metal and polymer-based substrates, positioning them as an ideal choice for the fabrication of microchannels employing these materials [115,116]. Figure 9 demonstrates a comparison of UV- and IR-based laser micromachining methods. CO_2_ lasers, positioned within the mid-IR range with a wavelength of 10,640 nm, have exhibited effectiveness in micromachining applications [117].

**Figure 10:**
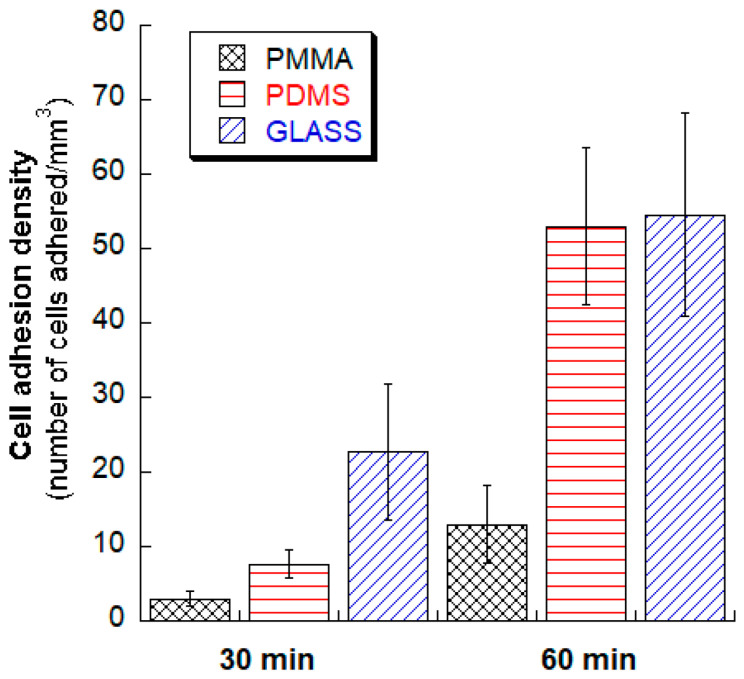
The cell adhesion density of each of the substrates (PDMS, PMMA, and glass) using laser micromachining for fabrication at incubation times of 30 and 60 min using the human fibroblast cell adhesion test [115].

**Figure 11:**
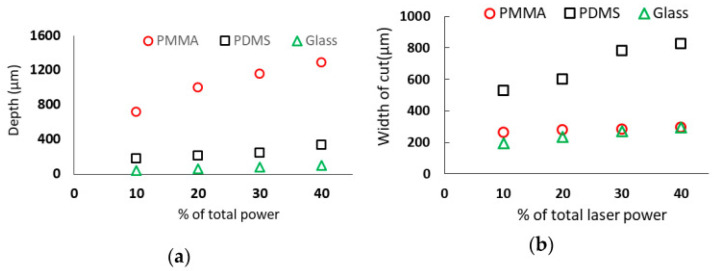
(**a**) Depth and (**b**) width of the channels of the three substrates (PDMS, PMMA, and glass) at different percentages of total power. The relationship of power to desired depth/width can be seen [115].

The utilization of CO_2_ laser micromachining represents a current trend in laser technology owing to its cost-effectiveness and remarkable versatility [116]. In a specific research study focused on the application of laser micromachining to widely used microfluidic materials, the investigators conducted experiments using CO_2_ laser micromachining on PDMS (polydimethylsiloxane), glass, and PMMA (polymethyl methacrylate). The use of a 45-Watt CO_2_ laser system yielded highly promising results, as depicted in Figure 10, which illustrates the cell adhesion density for each substrate, demonstrating the preeminence of laser micromachining as a fabrication method for microchannels in PDMS, PMMA, and glass. Following testing, Figure 11 was generated to visualize the relationships between the three substrate materials after cell adhesion incubation test times of 30 and 60 min using human fibroblast cells.

The experimental data analysis yielded important findings. First, an increase in laser power results in the growth of both the depth and width of a microchannel across all three substrates. Nevertheless, the widening of the channel in PMMA and glass substrates demonstrates a less significant increment compared to the increase in depth. When subjected to the same laser power, PMMA materials generated the deepest channel, while PDMS substrates produced the widest channel. Second, the maximum depth of cut and the minimum width of the cut occurs when substrates are positioned at the focal distance of the system for all three substrate materials, at a given laser parameter. Moreover, in all three substrates, the depth and width of the microchannel decrease with an increase in laser scanning speed. Also, elevating the number of passes leads to a more circular channel profile in glass and PDMS substrates, while in PMMA, it introduces a sharper corner at the channel’s bottom. Finally, PDMS exhibits greater sensitivity in cut width, while PMMA demonstrates greater sensitivity in cut depth for different combinations of laser system parameters. Glass, on the other hand, is more fragile during laser machining, resulting in increased breakage and the production of more fragmented edges in the machined microchannels [115]. The aforementioned conclusions demonstrate the efficacy of laser micromachining as a method for producing microchannels in microfluidic devices. This approach can be customized to suit specific device requirements, as it effectively retains the properties of the selected substrates.

In another study focused on the utilization of laser micromachining for the fabrication of PMMA microchannels, similar findings reinforced the idea that microfluidic systems in PMMA can be readily manufactured through CO_2_-laser micromachining. The findings indicated that the depth of microstructures can be regulated by the laser power, writing speed, and the number of laser beam passes, with a strong reliance on the material [116]. The ability of PMMA to achieve controlled depths through laser micromachining enables precise development and cost-effective mass production of microchannels. The growing adoption of PMMA (polymethyl methacrylate) as the substrate can be attributed to the rise of laser micromachining. The affordability of PMMA, combined with the mass production capability of laser micromachining, has significantly contributed to this trend [2].

In another recent investigation into the application of laser micromachining on EN31 Steel utilizing a confocal sensor, it was observed that the confocal sensor successfully provided real-time, accurate measurements of the dimensions of the microchannels during the laser micromachining process. This technique holds promising potential for manufacturing and quality control processes that necessitate the precise measurement of microchannels [120]. Once more, this study underscores the capability of laser micromachining to meticulously fabricate the microchannels essential for microfluidic devices across a range of substrates. These findings underscore the growing significance of laser micromachining as a fabrication technique in the realm of microfluidics. The advancement of using CO_2_ laser systems for the large-scale production of cost-effective, reproducible microfluidic devices indicates that the future of laser micromachining is imminent. Leveraging direct laser micromachining, microfluidic components can be integrated into contact lenses, thus expanding their functionalities in sampling, mixing, processing, and multiplexing [121].

Finally, we will discuss the categorization of laser micromachining techniques in microfabrication based on laser power and pulse duration, both of which significantly affect material removal, resolution, and surface morphology. High-power lasers facilitate rapid material ablation but may cause thermal damage and rough edges due to excess heat, while low-power lasers offer more controlled, precise fabrication with minimal heat-affected zones (HAZ), albeit at a slower pace. Pulse duration further refines these techniques. Femtosecond lasers, with their ultra-short pulses, minimize thermal diffusion, reduce heat-affected zones (HAZ), and produce superior precision, making them ideal for high-aspect-ratio microstructures with sub-micron features and minimal collateral damage [122,123]. In contrast, nanosecond lasers, though effective for micromachining polymers like PMMA, generate more HAZ due to longer pulse durations [124]. Studies by [125,126] further emphasize that adjusting laser pulse time and energy is key to optimizing micromachining for different materials and applications, balancing speed and precision for industrial and biomedical uses.

### 2.8. Injection Molding

**Figure 12:**
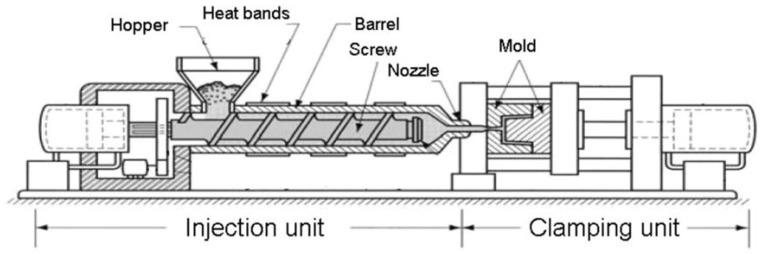
Different elements of an injection molding machine [128]. As depicted, the thermoplastic material is heated to a liquid state within the chamber. Simultaneously, the two halves of the mold are compressed to form the cavity. Subsequently, the molten thermoplastic is injected into the cavity through the nozzle. Finally, the mold is cooled, and the resulting microchannel is extracted from the machine.

The process of injection molding leverages a variety of thermoplastics to produce high throughput, cost-effective, and precise microfluidic devices [1]. Techniques such as injection molding and continuous reel-to-reel roller imprinting enable the completion of a replication cycle within seconds, making them well-suited for high-volume production [127]. Injection molding involves four steps to create microchannels for use in diverse microfluidic devices. Initially, the thermoplastic is precisely melted into a liquid state within a compressible chamber. Subsequently, the two halves of the mold are compressed, creating a mold cavity. The thermoplastic is then injected at a specific rate to fill the mold cavity. Finally, the mold is cooled, and the cast part is removed from the mold [1]. Figure 12 clearly indicates the different parts and steps of the injection molding machine.

Injection molding processes for microchannels are currently restricted to the use of thermoplastics like PMMA as the substrate due to specific constraints (such as mentioned in citations [1,129]). The complexity of designing undercuts in microchannels using injection molding limits the design options for injection molds, leading to more basic configurations. Ongoing research primarily centers on reducing the cost and time required for mold fabrication, as well as augmenting the functionality of existing methods and materials [1].

In an investigation pertaining to the application of injection molding on PMMA, a microfluidic device was developed, incorporating various common microfluidic components such as a single droplet generator, diffusion mixer, straight channels, and a double emulsion droplet generator. The analysis concluded that the use of PMMA for injection molding is notably effective, particularly in scenarios where mass production is feasible. However, it was observed that the initial cost of mold creation is substantial and justifiable only when mass replication is imperative [1,129]. Furthermore, the study identified potential defects, such as cracks and splays on the mold, associated with injection molding, emphasizing the significance of the initial cost in comparison to the benefits achieved once the mold is refined for mass production [130].

The process parameters, including the melt temperature, mold temperature, injection pressure, and holding pressure, significantly influence the final part’s quality, performance, and accuracy. In addition, the substrate material used also plays a crucial role in determining the process dynamics and output characteristics [131,132,133]. While PMMA is widely used for its optical clarity and ease of processing, other materials, such as polycarbonate (PC) and polypropylene (PP), polyethylene (PE), and acrylonitrile butadiene styrene (ABS), may be better suited for specific applications depending on the mechanical and thermal properties required. Polycarbonate excels in impact resistance, optical clarity, and thermal stability, making it ideal for electronics and lenses. Furthermore, polypropylene is lightweight, chemically resistant, and widely applied in packaging, medical devices, and automotive parts. Moreover, polyethylene, available as LDPE and HDPE, is tough and chemically resistant, used in containers and pipes. In addition, acrylonitrile butadiene styrene is tough, impact-resistant, and common in automotive and electronics [134].

In terms of temperature, two critical temperatures are essential in injection molding: 1. melt and 2. mold temperature. The melt temperature refers to the temperature at which the polymer is heated to transition into a flowable state. If the melt temperature is too low, the polymer may not fill the mold, leading to defects such as short shots. If it is too high, it can cause thermal degradation and compromise the polymer’s mechanical properties. The melt temperature of the PMMA, PC, PP, PE, and ABS is 200–250 °C, 260–310 °C, 160–250 °C, 160–250 °C, and 220–250 °C, respectively. On the other hand, the mold temperature controls how quickly the part cools and solidifies. A higher mold temperature improves surface finish but may increase cycle times. The mold temperature of the stated materials is 80 °C, 90–110 °C, 30–80 °C, 30–70 °C, and 50–70 °C, respectively [135,136,137].

Additionally, in terms of pressure, two primary pressures are effective in injection molding, including 1. injection and 2. holding pressure. The injection pressure is the force applied to inject molten material into the mold cavity. It is the initial pressure exerted to overcome the material’s resistance as it flows through the nozzle and into the mold. The injection pressure for PMMA, PP, PC, PE, and ABS is 60–120 MPa, 50–100 MPa, 90–150 MPa, 40–80 MPa, and 60–110 MPa, respectively. However, holding pressure is applied after the mold cavity has been filled but before the plastic solidifies. It compensates for material shrinkage as it cools and hardens, preventing voids, sink marks, and warping. It also ensures that the final part has accurate dimensions and smooth surfaces. Of note, it is usually lower than the injection pressure [138,139,140].

The future of injection molding is focused on minimizing the initial cost of mold creation and enhancing the functionality of thermoplastics [1,130]. Emphasis is also placed on reducing defects within molds, which, ultimately, minimizes the associated initial cost. A study on the deformation effects of injection-molded devices revealed that certain defects, such as stretching and fractures, occurred at the micro-structure edges of injection-molded microfluidic chips [141]. Ongoing research in injection molding aims to develop methods that reduce the time and cost involved in initial mold fabrication.

### 2.9. Micro CNC and Micro-Milling

The utilization of a micro CNC or micro-milling machine, which integrates computer numerical control technology, enables the precise cutting into small components using drills. To achieve optimal performance and quality during cutting, several pivotal setup factors, along with diverse materials and methods to enhance surface quality, must be considered. The spindle speed, feed rate, and depth of cut significantly influence the outcome during the cutting process. The precision of micro CNC holds paramount importance, especially considering its application in electronics, medical devices, and the fabrication of micro- and nanochannels [142]. For the creation of small channels, miniature milling tools eliminate a substrate with an accuracy range of tens of micrometers to a few millimeters for microfluidics [143]. Over the last five years, there have been notable advancements in micro CNC technology for fabricating micro- and nanochannels, with a continuous progression towards heightened precision and more intricate geometries.

In 2020, a novel fabrication technique combined CNC micro-milling with 3D printing to produce microfluidic devices designed for tumor spheroid generation. Researchers employed a stereo lithography 3D printer to fabricate the device from polycarbonate (a thermoplastic polymer) and, then, utilized CNC micro-milling to eliminate the channel substrates. Tumor spheroids, agglomerates of cancer cells used to investigate tumor growth and drug response, can be effectively studied through this method. The micro-milling process enables the creation of a more comprehensive 3D model compared to traditional 2D cell models, facilitating research into drug response and tumor growth [144]. This research yielded a microfluidic device capable of consistently generating uniform tumor spheroids. The integration of micro-milling allows for the large-scale production of these devices with exceptional precision.

The utilization of CNC micro-milling enables the study and live imaging of tumor spheroids within a microfluidic device. By synthesizing a nitrogen-sulfur co-doped carbon dot and introducing it to the microfluidic device containing the tumor spheroids, the researcher facilitated the diffusion of carbon dots into the spheroid to label the cells for imaging. A 3D cell culture microfluidic device offers improved nutrient gradient, hypoxia, and heat control. In this study, a microfluidic device with 57 microwells was designed and 3D printed, with the central channels in the chip produced using a CNC micro-mill. The resulting flow across the 57 wells is uniform, with simulations demonstrating a higher flow rate in the top layer, enabling the trapping and production of tumor spheroids. The use of CNC micro-milling empowered the researcher to engage in advanced laboratory research on a microfluidic chip that was both designed and constructed [145].

**Figure 13:**
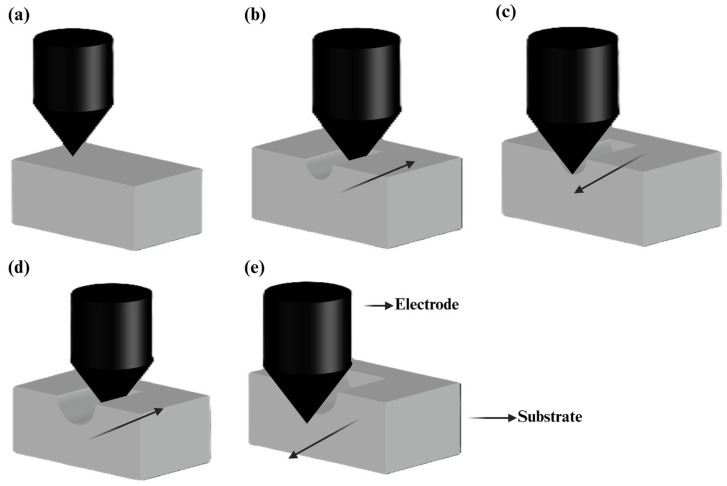
Schematic of a multi-pass micro-milling micromachine and its working principle. (**a**) The process begins with precise planning of the tool paths, determining the movement of the milling tool across the material to form the desired microchannel geometry. (**b**) The initial pass starts in the specified direction, making the first cut into the substrate. (**c**) The tool then reverses direction. (**d**) In subsequent passes, the too incrementally removes more material, gradually deepening the microchannel. This step-by-step approach helps to control the forces exerted on both the tool and the workpiece, minimizing the risk of tool deflection or breakage and ensuring the accuracy of the microchannel dimensions. (**e**) The tool completes a return pass as part of the multipass sequence (adapted from [146]).

Another method for creating micro- and nanochannels is the multi-pass electrochemical discharge-based micro-milling technique. In this approach, researchers employed a 50-micron tungsten carbide milling tool to apply a voltage, causing electrochemical discharge to remove material from glass. To create a precise substrate channel, multiple iterations of the tool are required to pass over the substrate area with an optimal applied voltage. The voltage level and size of the tool can be adjusted to achieve the necessary material removal rate, offering better results than micro CNC with a small drill bit [146]. Figure 13 illustrates the different stages of the multi-pass micro-milling micromachine. Conventional micro CNC presents challenges such as surface treatment, material quality, and drill bit wear and tear [142]. However, electrochemical discharge for microchannel fabrication allows for the creation of small channels when necessary, along with a high material removal rate. This innovative technology can greatly benefit the mass production of microfluidic chips made of glass [146].

## 3. Comparison of Fabrication Methods

The fabrication of micro- and nanochannels is crucial in various fields, including microfluidics, biomedical engineering, and nanotechnology. Table 1 provides a chronological comparative overview of several fabrication techniques, highlighting their performance in terms of resolution, cost, speed, complexity, and scalability, in addition to mentioning the introduction year of each method. Photolithography, widely used in semiconductor manufacturing, offers high resolution and scalability but comes at a high cost and complexity. Soft lithography, using PDMS, provides a cost-effective and flexible approach with moderate scalability and resolution in the sub-micron range. Techniques like electron-beam lithography (EBL) and focused ion beam (FIB) deliver resolutions in the sub-10 nm range, making them ideal for nanoscale features, but they suffer from very high costs, slow processing speeds, and limited scalability due to their serial nature. In contrast, more recent approaches like 3D printing offer a balance between moderate resolution and high scalability, making them a popular choice for customizable designs. Traditional etching techniques, both dry (RIE/DRIE) and wet, offer a range of resolutions and costs, with dry etching being suitable for large-scale production despite its complexity. Other methods, such as laser micromachining and injection molding, also provide fast processing speeds and scalability but are limited by factors like resolution and high initial setup costs, respectively. This comparison emphasizes that the choice of fabrication method depends heavily on the specific application, balancing the trade-offs between precision, cost, and scalability.

## 4. Future Applications

The application of micro- and nanochannels is set to have a significant impact not only on microfluidics for biomedical purposes but also on energy conservation. Microchannels can enhance the effectiveness of solar power refrigeration systems [147,148]. A membrane-based absorber is crucial for the refrigeration system as it comes into contact with the refrigerant vapor using an absorbent solution and is subsequently transferred to a generator [149,150]. The interaction between fluids determines the efficiency, and microchannels facilitate increased surface area contact to optimize heat transfer across the entire system. The high aspect ratio and small diameter of the channels enable superior performance across various climates, potentially increasing system efficiency by up to 20% [151]. Research into microchannels that contribute to energy conservation and reduced consumer electricity costs represents a pivotal area for producers seeking to minimize the environmental impact on consumers [152].

**Figure 14:**
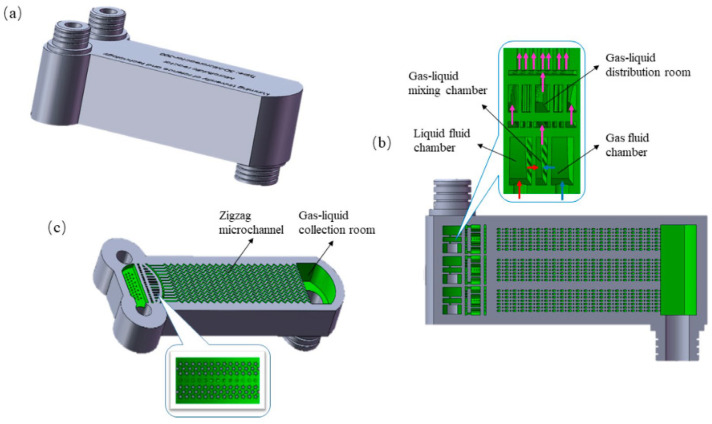
(**a**) 3D schematic and (**b**,**c**) profiles of the 3D-printed multichannel large-volume microchannel reactor primarily consisting of a gas fluid chamber, a liquid fluid chamber, a gas–liquid mixing chamber, a gas–liquid distribution chamber, a gas–liquid collection chamber, and numerous zigzag microchannels [156].

Microchannels incorporated into reactors offer a compelling solution to enhance air quality and mitigate environmental pollution [153,154]. Traditionally, micromachining has been employed in reactor production; however, 3D printing enables the mass production of microchannels, which effectively remove NO_x_ from flue gas that is emitted from industrial and power plant combustion and contains pollutants such as nitrogen oxides, carbon dioxide, and sulfur dioxide, posing detrimental effects on air quality and human health [155]. An example of 3D-printed reactors is demonstrated in Figure 14. The utilization of microchannels results in a higher surface area to volume ratio, leading to improved mass transfer and reduced gas-phase diffusion resistance. Additionally, the increased number of microchannels and their honeycomb design serves to enhance gas flow uniformity, further augmenting NO_x_ removal from the environment. As flue gas enters reactors with microchannels, the catalyst material facilitates the conversion of NO_x_ to nitrogen and water through selective catalytic reduction, achieving an efficiency of 90%. This study underscores the constructive impact of microchannel fabrication in creating a safer environment and planet for future generations [156].

Microchannels have practical applications in the realm of electronics for cooling purposes [157]. With the escalating energy consumption of electronic devices driven by heightened processing requirements, heat generation has the potential to impede performance or cause device malfunction [158]. The employment of microchannels, containing a coolant mixture of water and ethylene glycol, proves effective in absorbing heat from the device and transporting it away [159]. Analogous to the function of the reactor system, the material’s high surface area to volume ratio enables the catalyst to operate with greater efficiency [156]. In the context of electronic cooling, the catalyst takes the form of a fluid, functioning by circulating coolant, absorbing device heat via conduction, and dissipating it into the environment. This approach facilitates enhanced electronic computation by bolstering chip performance at an optimal temperature, thereby expanding the scope of consumer technology and enhancing performance as data complexity grows [160].

**Figure 15:**
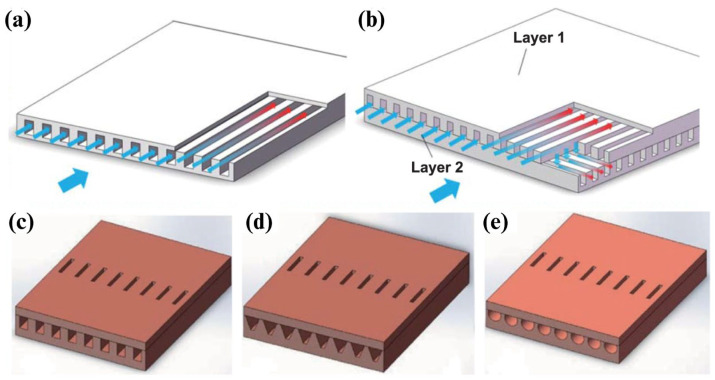
(**a**) The schematic of parallel MCHS microchannel consisting of channels designed for fluid flow. (**b**) Double-layer parallel microchannel. MCHS with different structures of microchannel including (**c**) rectangular, (**d**) trapezoidal, and (**e**) circular [161].

MCHS, or microchannel heat sinks, are highly efficient cooling devices used to dissipate heat from high-performance electronic components, such as microprocessors, power electronics, and laser diodes. They are particularly valuable in applications where managing heat is critical to maintaining performance, reliability, and longevity. Figure 15 shows the 3D schematic of a microchannel heat sink and different designs for the microchannels. MCHS consists of a series of parallel microchannels etched or machined into a solid substrate, typically made from materials with high thermal conductivity like copper, aluminum, stainless steel, or silicon [161,162]. MCHSs with varying channel cross-sections exhibit different levels of heat dissipation performance [163,164,165]. Despite extensive research, there is still no consensus among scientists on which microchannel shape delivers the best performance. Some studies revealed the performance of rectangular microchannels [166], and another study [167] showed that triangular-shaped microchannels tend to be better than the other proposed channel structures.

## 5. Conclusions

In conclusion, the technological advancements in the fabrication of micro- and nanochannels mark a pivotal shift in how these critical structures are designed and produced. From traditional methods like photolithography and soft lithography to newer approaches such as 3D printing and laser micromachining, each technique has demonstrated unique strengths in terms of resolution, scalability, and cost-effectiveness. These innovations not only address the inherent limitations of older methods but also open up new possibilities for applications in biomedical research, diagnostics, and environmental monitoring. Future developments in micro- and nanochannel fabrication will likely focus on enhancing the precision and reducing the cost of production, particularly in mass manufacturing. As the field continues to evolve, these emerging technologies promise to play a crucial role in shaping the next generation of microfluidic devices, leading to improved performance, broader adoption, and more innovative applications.

## Figures and Tables

**Table 1:** Comparison of fabrication methods, listed from oldest to most recent, based on key factors and years of usage.

Method	Resolution	Cost	Speed	Complexity	Scalability	Year Introduced
Wet Etching [86,87,88,89,90]	Medium (micron scale)	Low	Fast	Simple	Low; limited by undercutting issues	1950s
Photolithography [43,44]	~100 nm	High	Moderate	High	High; good for large-scale production	1960s
Electron Beam Lithography (EBL) [61,62,63,64,65,66,67,68]	Sub-10 nm	Very High	Very Slow	Very High	Low; due to serial nature and high cost	1970s
Focused Ion Beam (FIB) Milling [84,85]	Sub-10 nm	Very High	Slow	High	Low; limited by precision and cost	1980s
Dry Etching (RIE/DRIE) [92,93,94,95]	High (nanoscale)	High	Medium	Complex	High; suitable for large-scale production	1980s
Injection Molding [1,127,128,129,130,141]	Low (micron scale)	High (initial cost)	Fast (after mold creation)	Simple once set up	Very high; suitable for mass production	1980s
Soft Lithography (with PDMS) [49,50,51,52,53,54,55,56,57]	~10 nm to 1 µm	Low	Fast	Low	Moderate; flexible and scalable	1990s
Laser Micromachining [115,116,117,118,119,120,121]	Medium (micron scale)	Moderate to High	Fast	Moderate	Medium; limited by laser resolution	1990s
Micro CNC or Micro-milling [142,143,144,145,146]	~10 µm to 1 mm	Moderate	Moderate to Slow	High	Moderate to high; suitable for mass production	1990s
3D Printing [96,97,98,99]	~10 µm	Low toModerate	Fast	Low to Moderate	Moderate to high; customizable and scalable	2010s

## Data Availability

No new data were created or analyzed in this study. Data sharing is not applicable to this article.
